# The association of novel inflammatory marker GlycA and incident atrial fibrillation in the Multi-Ethnic Study of Atherosclerosis (MESA)

**DOI:** 10.1371/journal.pone.0248644

**Published:** 2021-03-25

**Authors:** Sunyoung Jang, Oluseye Ogunmoroti, Di Zhao, Oluwaseun E. Fashanu, Martin Tibuakuu, Eve-Marie Benson, Faye Norby, James D. Otvos, Susan R. Heckbert, Moyses Szklo, Erin D. Michos

**Affiliations:** 1 Ciccarone Center for the Prevention of Cardiovascular Disease, Johns Hopkins University School of Medicine, Baltimore, Maryland, United States of America; 2 Department of Epidemiology, Johns Hopkins Bloomberg School of Public Health, Baltimore, Maryland, United States of America; 3 Department of Medicine, Saint Agnes Hospital, Baltimore, Maryland, United States of America; 4 Division of Epidemiology and Community Health, University of Minnesota School of Public Health, Minneapolis, Minnesota, United States of America; 5 Laboratory Corporation of America Holdings, Morrisville, North Carolina, United States of America; 6 Department of Epidemiology, University of Washington, Seattle, Washington, United States of America; German Centre for Neurodegenerative Diseases Site Munich: Deutsches Zentrum fur Neurodegenerative Erkrankungen Standort Munchen, GERMANY

## Abstract

**Background:**

Emerging evidence has implicated that inflammation contributes to the pathogenesis of atrial fibrillation (AF). GlycA is a novel marker of systemic inflammation with low intra-individual variability and high analytic precision. GlycA has been associated with incident cardiovascular disease (CVD) independent of other inflammatory markers. However, whether GlycA is associated with AF, specifically, has yet to be established. We examined the association between GlycA and AF in a multi-ethnic cohort.

**Methods:**

We studied 6,602 MESA participants aged 45–85, with no clinical CVD at baseline, with data on GlycA and incident AF. We used multivariable-adjusted Cox models to evaluate the association between GlycA and incident AF. We also examined other inflammatory markers [high-sensitivity C-reactive protein (hsCRP), interleukin-6 (IL6) and fibrinogen] and incident AF for comparison.

**Results:**

The mean (SD) age was 62 (10) years, 53% women. The mean plasma GlycA was 381 (62) μmol/L. Over median follow-up of 12.9 years, 869 participants experienced AF. There was no statistically significant association between GlycA and incident AF after adjusting for sociodemographics, CVD risk factors, and other inflammatory markers [Hazard Ratio (95% CI) per 1 SD increment in GlycA: 0.97 (0.88–1.06)]. Neither hsCRP nor fibrinogen was associated with incident AF in same model. In contrast, IL-6 was independently associated with incident AF [HR 1.12 per 1 SD increment (1.05–1.19)].

**Conclusions:**

Although GlycA has been associated with other CVD types, we found that GlycA was not associated with AF. More research will be required to understand why IL-6 was associated with AF but not GlycA.

**Clinical trial registration:**

MESA is not a clinical trial. However, the cohort is registered at: URL: https://clinicaltrials.gov/ct2/show/NCT00005487 Unique identifier: NCT00005487.

## Background

Atrial fibrillation (AF) is the most common type of cardiac arrhythmia, characterized by chaotic atrial electrical signals leading to irregular heart rhythm [1, 2]. AF, which has affected more than 30 million people worldwide since 2010, occurs in 1–2% of the general population <65 years of age, but ~9% of those over the age of 65 [3, 4]. Regarded as a chronic illness, AF is associated with compromised quality of life and deleterious consequences including dementia, heart failure, and cardiogenic stroke from embolic events [1]. Therefore, prevention of AF and identification of risk are paramount. Many traditional risk factors such as obesity, hypertension, diabetes, smoking, and coronary artery disease (CAD) are well-known risk factors for AF. However, the methods available for predicting incident AF risk and establishing the underlying pathophysiology are still limited.

Many of the same chronic systemic diseases that are linked to AF (i.e. CAD, hypertension, and obesity) are also associated with persistent low-grade inflammation and increased levels of proinflammatory cytokines [5, 6]. There has been increased recognition of inflammation as a central mediator of AF in hearts that are metabolically stressed [7–9]. The prevalence and prognosis of AF are both associated with serum levels of circulating inflammatory biomarkers and cardiac tissue expression of inflammatory markers [2, 10, 11]. Inflammation exacerbates electrical and structural remodeling of the atrium leading to the formation of an adverse substrate that facilitates onset and maintenance of AF [12]. Moreover, AF itself can induce inflammation during atrial remodeling, thereby perpetuating the arrhythmia [2]. Inflammatory markers, such as interleukin 6 (IL-6), high-sensitivity C-reactive protein (hsCRP), and fibrinogen have been demonstrated to be associated with AF [13–16].

GlycA, an emerging biomarker of systemic inflammation, which is a nuclear magnetic resonance (NMR) signal derived from the *N*-acetyl methyl group protons of circulating glycosylated proteins: α1-acid glycoprotein, α1-antitrypsin, haptoglobin, α1-antichymotrypsin and transferrin [17]. GlycA is moderately correlated with other known markers for inflammation, such as hsCRP, IL-6, and fibrinogen [18]. Yet, compared to these other common inflammatory markers, GlycA has low intra-individual variability and greater analytic precision, which makes it a more reliable inflammatory biomarker [17]. Moreover, elevated GlycA has been associated with incident cardiovascular events, such as myocardial infarction, ischemic stroke, coronary revascularization, peripheral artery disease, heart failure, and CVD death, independent of other traditional inflammatory markers [19–23]. However, whether GlycA is associated with AF, specifically, has not been well established. This is important to evaluate because GlycA may be a prognostic marker for AF or a target of therapy in the future.

To address this knowledge gap, we sought to determine the association of baseline GlycA and AF in a large multi-ethnic cohort free of overt clinical CVD at baseline. We hypothesized that higher plasma levels of baseline GlycA are associated with increased incidence of AF, independent of other traditional risk factors and other common inflammatory markers.

## Material and methods

### Transparency and Openness Policy (TOP)

The Multi-Ethnic Study of Atherosclerosis (MESA) participates in the NIH BioLincc Open program, and requests for access to MESA data can be submitted to: https://biolincc.nhlbi.nih.gov/studies/mesa/. Additionally, other researchers can apply to the MESA Coordinating Center to become a new investigator after signing a Data Use Agreement (DUA); more details are available at https://www.mesa-nhlbi.org/. Upon receipt of a DUA with the MESA Coordinating Center, access to the de-identified MESA databases can be made available to other researchers with approved proposals. Our findings should be easily reproducible through the methods described in this paper.

### Study sample

MESA is an ongoing prospective cohort study that recruited ethnically diverse individuals free of clinically recognized CVD, including AF, and apparently healthy at baseline from 6 field centers in the United States to assess the characteristics of subclinical CVD, and risk factors for its progression. According to the MESA study protocol, individuals with clinically recognized CVD were excluded from the study. This refers to people who had physician-diagnosed heart attack, angina, stroke, transient ischemic attack, heart failure, or atrial fibrillation, those taking nitroglycerin and those who underwent procedures related to CVD (coronary artery bypass grafting, angioplasty, valve replacement, pacemaker or defibrillator implantation, any surgery on the heart or arteries).

The study initially enrolled a total of 6,814 individuals aged 45 to 84 years, 38% White, 28% African, 22% Hispanic, and 12% Asian between 2000 and 2002. Since the initial visit, 5 subsequent visits have taken place. At each visit, participant demographics, medical history, and physical examination results were collected. Further details about the study design have been reported [24].

**Fig 1:**
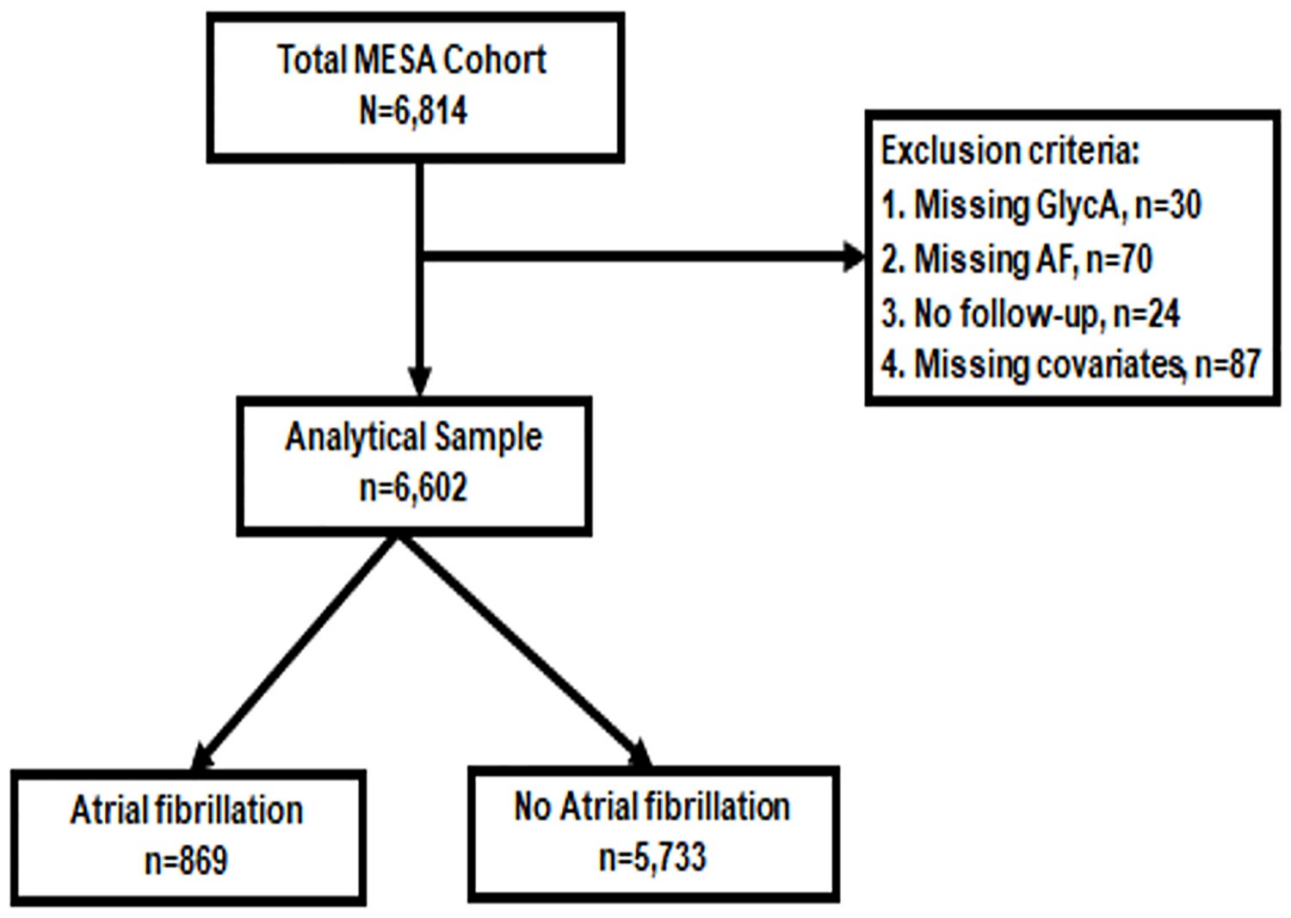
Flow diagram illustrating study sample inclusion and exclusion criteria as well as the number of participants with and without AF during follow-up.

In our analysis, individuals missing either GlycA measurements at baseline or incident AF follow-up data were excluded, leaving a total of 6,602 participants for inclusion (Fig 1).

### Ethical approval statement

The MESA study was approved by the institutional review boards (IRB) at each participating center and each study participant provided informed consent. At Johns Hopkins, our study was approved by the Johns Hopkins School of Medicine IRB applicant number NA_00030361.

### Exposure assessment

GlycA was analyzed from EDTA plasma samples, which were obtained from MESA baseline visit (2000–2002) and stored at -70°C until measured using NMR LipoProfile^®^ as previously described [21, 23, 25]. The NMR assay detects the level of glycan residues of acute phase proteins and quantifies GlycA levels, which are expected to be elevated during inflammation [17]. The intra-assay and inter-assay coefficients of variance (CV) for NMR LipoProfile^®^ were 1.9% and 2.6%, respectively. GlycA levels haven been shown to be similar when measured after short-term vs. long-term storage, in plasma vs. serum samples, and fasting vs. non-fasting state [17].

### Covariates

Using data obtained from the baseline MESA visit, we considered demographics (age, sex, race/ethnicity, and MESA site), behavioral factors (smoking status, pack-years of smoking, and physical activity), socioeconomic factors (education, health insurance), adiposity [Body Mass Index (BMI)], traditional CVD risk factors [systolic blood pressure (SBP), use of antihypertensive medication, total cholesterol, HDL-cholesterol, use of lipid-lowering medication, diabetes and estimated glomerular filtration rate (eGFR)], and other inflammatory markers (hsCRP, IL-6, fibrinogen) for covariate adjustment. Height, weight, and blood pressure were measured following the protocol. BMI was calculated as weight divided by squared height (kg/m^2^). Physical activity was estimated in metabolic equivalent minutes per week using a 28-item Typical Week Physical Activity Survey [26]. Baseline blood pressure was measured 3 times in the seated position with the Dinamap automated blood pressure device, and the average of the last 2 measurements were used. Glomerular filtration rate was estimated using the formula from the Chronic Kidney Disease Epidemiology Collaboration [27]. Diabetes was defined as fasting blood glucose level ≥126 mg/dl, self-reported diagnosis of diabetes, or use of diabetes medication. Inflammatory biomarkers, including hsCRP, IL-6, and fibrinogen were measured from stored serum samples obtained at the baseline examination [18, 28].

### Outcomes assessment

Prevalent AF identified by study visit electrocardiogram (ECG) or self-report at baseline was an exclusion criterion for enrollment in the study. After enrollment, trained staff called study participants or their proxy every 9 to 12 months to identify all new hospitalizations and obtained discharge diagnostic codes. Participants were followed until July 15, 2015. Incident AF was ascertained by 1) study ECGs at a follow-up visit consistent with AF; 2) hospital discharge diagnoses [International Classification of Diseases, Ninth Revision, Clinical Modification code 427.31 (AF) or 427.43 (atrial flutter)] for AF; or 3) Medicare inpatient and outpatient claims data for individuals enrolled in fee-for-service Medicare. Previous work has shown the validity (up to 89%) of discharge diagnoses for identifying AF [29].

### Statistical analysis

Baseline GlycA, hsCRP, IL-6, and fibrinogen levels were modeled in quartiles and also continuously per one standard deviation (SD) increment. Baseline characteristics of study population were presented by incident AF status. Kaplan-Meier curves were plotted of AF-free survival by GlycA quartiles. Multivariable-adjusted Cox proportional hazard regression models were used to estimate hazard ratios (HRs) and their 95% confidence intervals (CIs) for the association of incident AF by GlycA levels.

We evaluated 4 progressively adjusted models. In model 1, we adjusted for demographics and study site (age, sex, race/ethnicity, MESA site). Model 2 adjusted for model 1 covariates with the addition of socioeconomic, behavioral, and adiposity measures (education, health insurance, smoking status, pack-years of smoking, physical activity, and BMI). Model 3 additionally adjusted for CVD risk factors that may be intermediate variables between inflammation/adiposity and AF risk (SBP, use of antihypertensive medication, total cholesterol, HDL-cholesterol, use of lipid-lowering medication, diabetes and eGFR). In model 4, we further adjusted for other commonly studied inflammatory markers, specifically log-transformed hsCRP, IL-6, and fibrinogen. In the same fully adjusted model (model 4), we also evaluated the association of the other inflammatory markers with incident AF, to compare with GlycA. In a supplemental analysis, we further adjusted for incident heart failure and incident coronary heart disease (CHD) in addition to all covariates adjusted for in model 3.

In addition, we used restricted cubic spline adjusted for the variables in Model 4 with knots placed at the 5th, 35th, 66.5th, 95^th^ percentiles to characterize the nonlinear association between baseline GlycA (continuous) levels with incident AF. We also examined multiplicative interactions of baseline GlycA (and the other inflammatory markers) with AF by age, sex, and race/ethnicity categories. The analyses were performed using STATA version 15.0 (StataCorp LP, College Station, TX). P values were two-sided, with significance level set at 0.05.

## Results

### Baseline characteristics

The baseline characteristics of the 6,602 participants included in the analyses are shown in Table 1 by incident AF status and S1 Table by baseline GlycA level quartiles. Among the sample, the mean (SD) for age was 62 (10) years with 53% being women, 38% White, 27% Black, 22% Hispanic, and 12% Chinese. The mean (SD) for baseline plasma GlycA level was 381 (62) μmol/L. Those with higher GlycA levels tended to be women, have a lower educational background, and have a higher BMI, SBP, and prevalence of diabetes mellitus. In addition, those with higher GlycA levels tended to have a higher median level of inflammatory biomarkers, such as hsCRP, IL-6, and fibrinogen **(**S1 Table).

**Table 1:** Baseline characteristics of participants by presence or absence of incident atrial fibrillation, multi-ethnic study of atherosclerosis (2000–2015).

	Total	AF	No AF	P value
N	6,602	869 (13%)	5,733 (87%)	
^a^ Age, years	62 (10)	69 (8)	61 (10)	< 0.001
< 65 years	3,739 (57%)	219 (25%)	3,520 (61%)	< 0.001
≥ 65 years	2,863 (43%)	650 (75%)	2,213 (39%)	
Sex				
Women	3,484 (53%)	391 (45%)	3,093 (54%)	
Race/ethnicity				
White	2,541 (38%)	411 (47%)	2,130 (37%)	< 0.001
Chinese American	791 (12%)	107 (12%)	684 (12%)	
Black	1,804 (27%)	190 (22%)	1,614 (28%)	
Hispanic	1,466 (22%)	161 (19%)	1,305 (23%)	
Education				0.92
< bachelor’s degree	4,267 (65%)	563 (65%)	3,704 (65%)	
^a^ BMI, kg/m^2^	28 (5)	28 (5)	28 (5)	0.68
Current smoker	855 (13%)	94 (11%)	761 (13%)	< 0.001
Former smoker	2,423 (37%)	374 (43%)	2,049 (36%)	
Never smoker	3,324 (50%)	401 (46%)	2,923 (51%)	
^b^ Pack-years of smoking if >0, median (IQR)	16 (6–33)	23 (9–42)	16 (6–31)	< 0.001
^b^ Physical activity, MET-minutes/week	4,035	3,435	4,170	< 0.001
	(1,995–7,545)	(1,650–6,413)	(2,040–7,740)	
^a^ Systolic blood pressure, mmHg	126 (22)	134 (22)	125 (21)	< 0.001
^a^ eGFR, ml/min per 1.73m^2^	74 (16)	71 (18)	75 (16)	< 0.001
^a^ Total cholesterol, mg/dL	194 (35)	191 (35)	195 (35)	0.005
^a^ HDL-C, mg/dL	51 (15)	51 (15)	51 (15)	0.53
Diabetes mellitus	820 (12%)	140 (16%)	680 (12%)	< 0.001
Antihypertensive medication	2,435 (37%)	443 (51%)	1,992 (35%)	< 0.001
Lipid-lowering medication	1,076 (16%)	187 (22%)	889 (16%)	< 0.001
^b^ GlycA, μmol/L	376 (337–420)	376 (338–417)	376 (337–421)	0.94
^b^ hsCRP, mg/L	1.91 (0.84–4.23)	1.92 (0.89–4.18)	1.91 (0.82–4.24)	0.67
^b^ IL-6 pg/mL	1.20 (0.77–1.88)	1.43 (0.92–2.17)	1.17 (0.75–1.84)	< 0.001
^b^ Fibrinogen, mg/dL	338 (295–388)	341 (304–392)	337 (294–388)	0.003

Abbreviations: AF, atrial fibrillation; BMI, body mass index; MET, metabolic equivalent of task; eGFR, estimated glomerular filtration rate; HDL-C, high-density lipoprotein cholesterol; hsCRP, high-sensitivity C-reactive protein; IL-6, interleukin-6.

^a^ Data are presented as mean (standard deviation) for continuous variables and as count (percentages) for categorical variables, unless otherwise specified.

^b^ Data are presented as median (IQR).

Over a median (IQR) follow-up time of 12.9 (10.0–13.6) years, a total of 869 participants (13%) experienced AF. Those who developed AF tended to be men, White, and have a higher age, median pack-years of smoking, SBP, and a greater prevalence of diabetes mellitus, and were more likely to take medications for hypertension and hyperlipidemia. They had lower average physical activity levels and eGFR, although BMI was similar to those who did not develop AF. Participants with AF had similar levels of baseline GlycA, hsCRP, and fibrinogen; however, baseline IL-6 levels were higher among those who developed incident AF (Table 1).

### Associations of GlycA with incident AF

**Fig 2:**
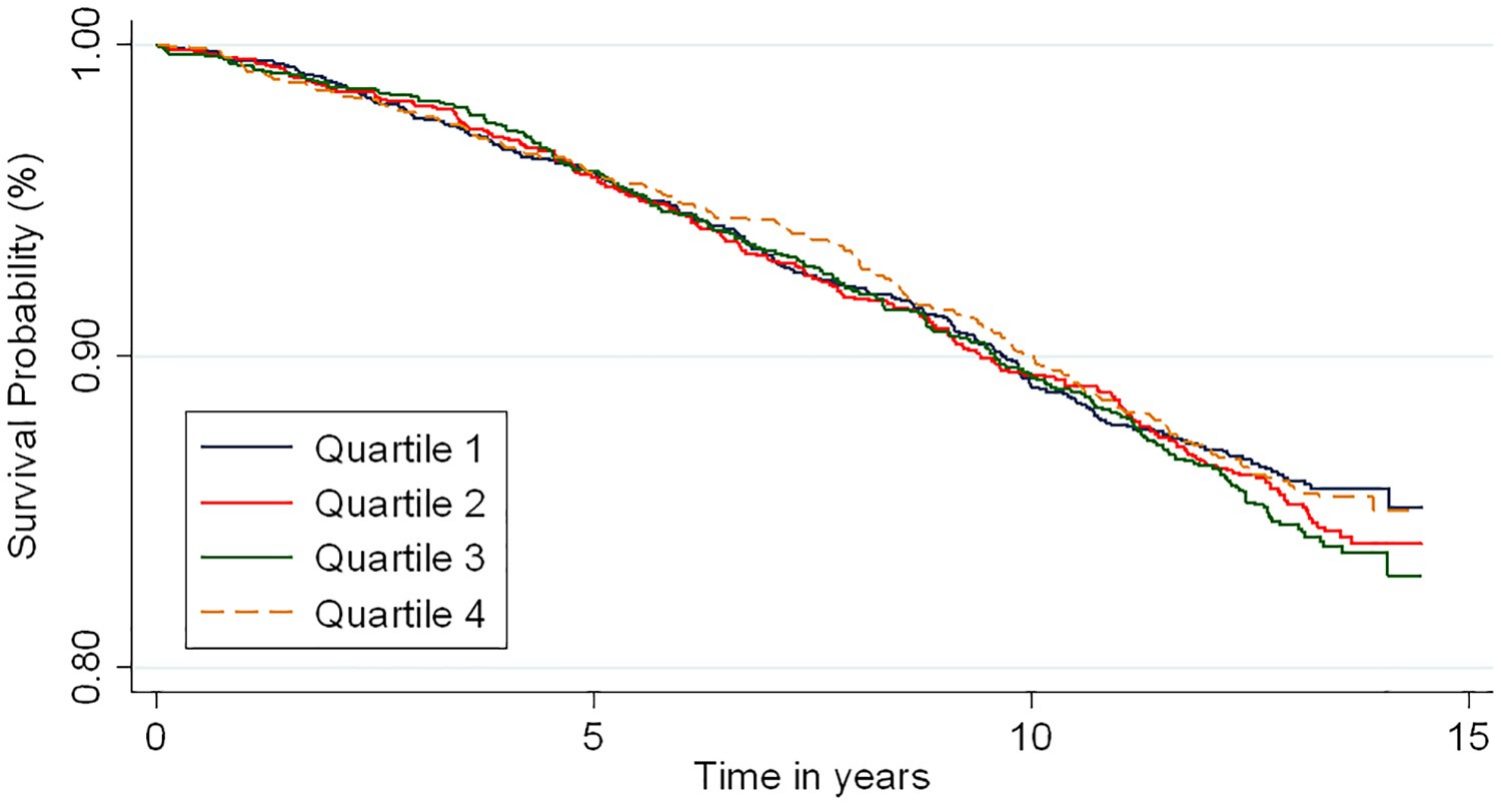
Kaplan-Meier survival estimates for the association between GlycA and atrial fibrillation. Log-rank test: P = 0.64.

**Fig 3:**
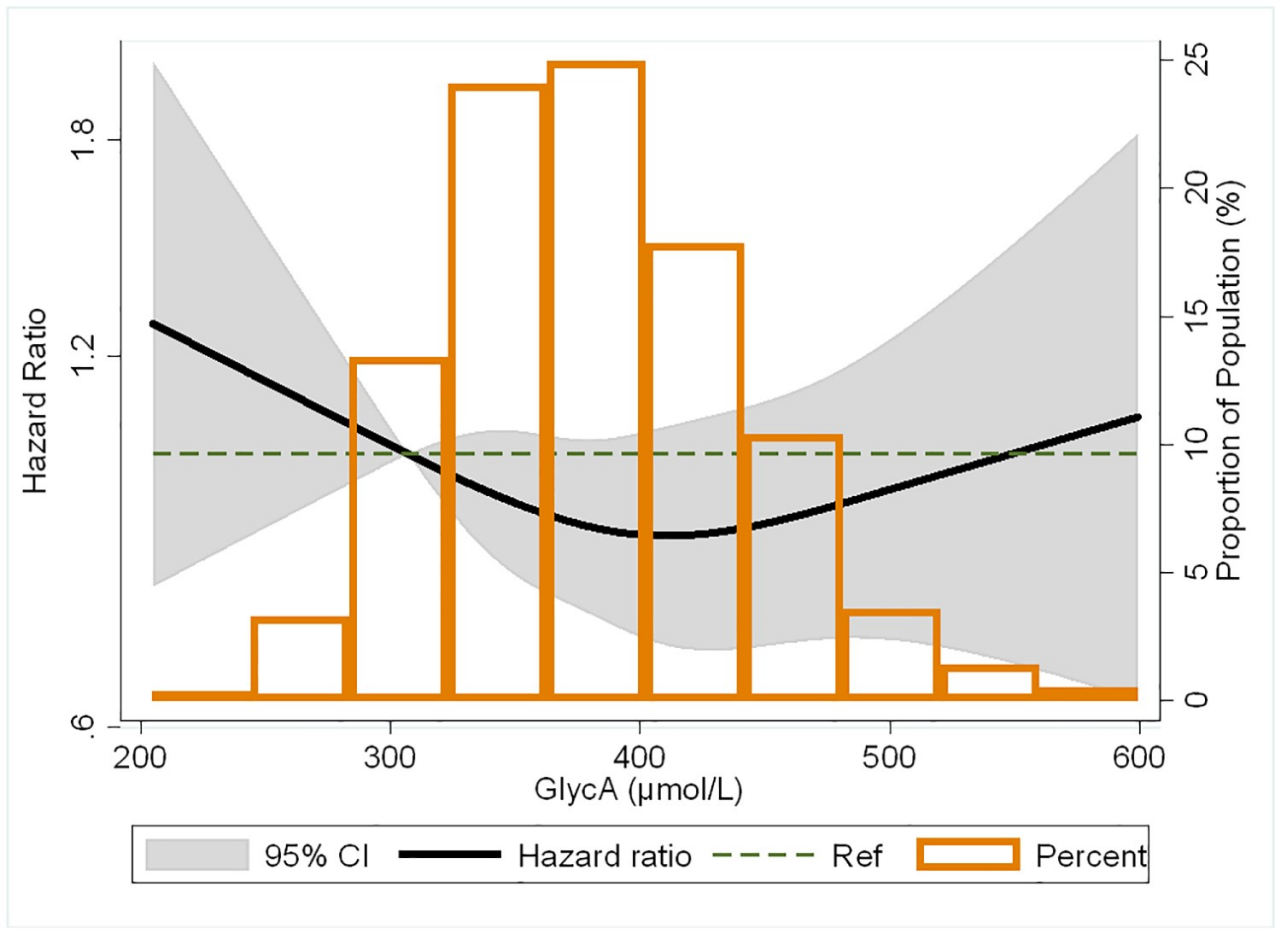
Restricted cubic spline of the association between GlycA and atrial fibrillation adjusted for age, sex, race/ethnicity, MESA field site, education, health insurance, BMI, smoking status, pack-years of smoking, physical activity, systolic blood pressure, use of antihypertensive medication, total cholesterol, HDL-cholesterol, use of lipid-lowering medication, diabetes, eGFR, ln(CRP), ln(IL-6) and ln(fibrinogen). The black curve represents hazard ratios for atrial fibrillation by proportion of population with the respective GlycA concentration. Grey boundaries represent the 95% CI of the hazard ratios. Knots were at the 5^th^, 35^th^, 66.5^th^, and 95^th^ percentiles which correlates to GlycA levels of 289.8, 353.1, 400.2, and 488.8 μmol/L, respectively.

The incidence rates and HRs for the association of baseline GlycA with incident AF are shown in Table 2. In unadjusted analysis, there was no significant difference in survival free of AF by baseline GlycA quartiles (Fig 2, log-rank test: P = 0.64). There remained no significant association of GlycA with incident AF in multivariable adjusted models. After adjusting for demographics, such as age, sex, race/ethnicity, and MESA site (model 1), compared to the lowest GlycA quartile, the adjusted HRs (95% CI) for AF were 1.03 (0.85–1.24), 1.11 (0.92–1.34) and 1.13 (0.92–1.38) for the 2^nd^, 3^rd^ and 4^th^ quartiles respectively. There also was no statistically significant association after progressively adjusting for behavioral, socioeconomic and adiposity factors (model 2), CVD risk factors that may be intermediate variables between inflammation/adiposity and AF risk (model 3), and other biomarkers for inflammation (model 4). Findings were similar with modeled continuously per 1 SD increment in baseline GlycA. There was no significant interactions by sex (p = 0.05), age (p = 0.65) or race/ethnicity (p = 0.23) for the association of baseline GlycA and incident AF. In the restricted cubic spline model, the association of baseline GlycA with risk of AF appeared U-shaped; however, confidence intervals were wide and associations not statistically significant (Fig 3).

**Table 2:** Incidence rates (95% CI) and hazard ratios (95% CI) for the association of GlycA with incident atrial fibrillation: The multi-ethnic study of atherosclerosis (2000–2015).

GlycA in μmol/L	Quartile 1	Quartile 2	Quartile 3	Quartile 4	Per 1 SD (62 μmol/L) increment
[Median (IQR)]	[314 (294–327)]	[359 (348–368)]	[397 (386–407)]	[452 (435–480)]	
N	1,670	1,661	1,630	1,641	6,602
Cases	213	226	228	202	869
Incidence rates†	11.2 (9.8–12.8)	12.0 (10.5–13.7)	12.4 (10.9–14.1)	11.2 (9.8–12.9)	11.7 (11.0–12.5)
Hazard Ratios*					
Model 1	1 (reference)	1.03 (0.85–1.24)	1.11 (0.92–1.34)	1.13 (0.92–1.38)	1.07 (0.99–1.15)
Model 2	1 (reference)	0.98 (0.81–1.19)	1.00 (0.83–1.22)	0.98 (0.79–1.21)	1.02 (0.94–1.10)
Model 3	1 (reference)	0.96 (0.79–1.17)	0.98 (0.81–1.20)	0.95 (0.77–1.18)	1.01 (0.93–1.09)
Model 4	1 (reference)	0.95 (0.78–1.16)	0.95 (0.77–1.17)	0.86 (0.67–1.10)	0.97 (0.88–1.06)

Abbreviation: CI, confidence interval; SD, standard deviation.

^†^ Crude incidence rates reported are per 1000 person-years.

*Model 1: Adjusted for age, sex, and race/ethnicity and MESA site.

*Model 2: Model 1 plus education, health insurance, BMI, smoking status, pack-years of smoking, and physical activity.

*Model 3: Model 2 plus systolic blood pressure, use of antihypertensive medication, total cholesterol, HDL-cholesterol, use of lipid-lowering medication, diabetes and eGFR.

*Model 4: Model 3 plus ln(CRP), ln(IL-6) and ln(Fibrinogen).

^‡^ P value = 0.65, 0.05 and 0.23 for interaction by age, sex and race/ethnicity using quartiles of GlycA as the exposure variable. Results remained similar and not statistically significant when stratified by sex.

Given that GlycA has already been demonstrated to be associated with incident heart failure and CHD, we performed a supplemental analysis to determine whether GlycA was still associated with AF after adjusting for interim heart failure event or CHD in addition to all covariates adjusted for in model 3 (S2 Table). In this supplemental analysis, the adjusted HR (95% CI) for AF per 1 SD higher GlycA was 0.96 (0.89–1.04), still showing no statistically significant association between GlycA and AF.

### Associations of hsCRP, IL-6, and fibrinogen with incident AF

Incidence rates and HRs for the association of other inflammatory biomarkers at baseline, such as hsCRP, IL-6, and fibrinogen with incident AF were also evaluated among the 6,446 individuals with complete biomarker assessment (Table 3). There was no significant association of hsCRP with incident AF with adjusted HRs (95% CI) of 1.00 (0.82–1.22), 0.94 (0.76–1.16) and 0.98 (0.76–1.26) for the 2^nd^, 3^rd^ and 4^th^ quartiles, respectively, compared to the first hsCRP quartile, in our fully adjusted model (model 4). Similarly, there was no association of fibrinogen with incident AF. Compared to the lowest fibrinogen quartile, the adjusted HRs (95% CI) for AF were 1.20 (0.98–1.47), 0.96 (0.78–1.19) and 0.94 (0.74–1.20) for the 2^nd^, 3^rd^ and 4^th^ quartiles respectively. On the other hand, higher IL-6 was independently associated with AF. Compared to the lowest IL-6 quartile, the adjusted HRs (95% CI) for AF were 1.05 (0.84–1.32), 1.33 (1.06–1.67) and 1.42 (1.10–1.82) for the 2^nd^, 3^rd^ and 4^th^ quartiles respectively, and was 1.12 (1.05–1.19) per 1 SD increment in IL-6. Interactions by age, race/ethnicity and sex were not significant for hsCRP, IL-6 and fibrinogen.

**Table 3:** Incidence rates (95% CI) and hazard ratios (95% CI) for the association of CRP, IL-6 and fibrinogen with incident atrial fibrillation: The multi-ethnic study of atherosclerosis (2000–2015).

CRP in mg/L	Quartile 1	Quartile 2	Quartile 3	Quartile 4	Per 1 SD
[Median (IQR)]	[0.5 (0.3–0.7)]	[1.3 (1.0–1.5)]	[2.8 (2.3–3.4)]	[7.3 (5.4–11.5)]	(5.2 mg/L) increment
N	1,617	1,614	1,604	1,611	6,446
Cases	195	230	216	207	848
Incidence rates†	10.5 (9.1–12.1)	12.6 (11.1–14.4)	12.1 (10.6–13.8)	11.5 (10.1–13.2)	11.7 (11.0–12.5)
Hazard Ratios*	1 (reference)	1.00 (0.82–1.22)	0.94 (0.76–1.16)	0.98 (0.76–1.26)	0.99 (0.91–1.08)
IL-6 in pg/mL	Quartile 1	Quartile 2	Quartile 3	Quartile 4	Per 1 SD
[Median (IQR)]	[0.6 (0.5–0.7)]	[1.0 (0.9–1.1)]	[1.5 (1.3–1.7)]	[2.7 (2.2–3.6)]	(1.2 pg/mL) increment
N	1,612	1,611	1,612	1,611	6,446
Cases	140	188	256	264	848
Incidence rates†	7.2 (6.1–8.5)	10.1 (8.7–11.6)	14.5 (12.8–16.4)	15.7 (13.9–17.7)	11.7 (11.0–12.5)
Hazard Ratios*	1 (reference)	1.05 (0.84–1.32)	1.33 (1.06–1.67)	1.42 (1.10–1.82)	1.12 (1.05–1.19)
Fibrinogen in mg/dL	Quartile 1	Quartile 2	Quartile 3	Quartile 4	Per 1 SD
[Median (IQR)]	[269 (250–284)]	[317 (307–327)]	[362 (349–373)]	[428 (405–463)]	(72 mg/dL) increment
N	1,643	1,583	1,638	1,582	6,446
Cases	177	230	223	227	868
Incidence rates†	9.3 (8.0–10.8)	12.9 (11.3–14.7)	12.1 (10.6–13.8)	12.6 (11.0–14.4)	11.7 (11.0–12.5)
Hazard Ratios*	1 (reference)	1.20 (0.98–1.47)	0.96 (0.78–1.19)	0.94 (0.74–1.20)	0.99 (0.91–1.08)

Abbreviation: CI, confidence interval; SD, standard deviation.

Sample size = 6,446 after excluding participants with missing data on IL-6 and Fibrinogen.

^†^ Crude incidence rates reported are per 1000 person-years.

*Model: Adjusted for age, sex, race/ethnicity, MESA site, education, health insurance, BMI, smoking status, pack-years of smoking, physical activity, systolic blood pressure, use of antihypertensive medication, total cholesterol, HDL-cholesterol, use of lipid-lowering medication, diabetes and eGFR and log transformed CRP, IL-6, fibrinogen, and including GlycA. Interactions by age, race and sex were not significant for CRP, IL-6 and Fibrinogen using quartiles of each inflammatory biomarker as the exposure variable.

## Discussion

Contrary to our hypothesis, in this analysis from a diverse community cohort free of CVD at baseline, we found that higher concentrations of a novel composite inflammatory biomarker GlycA at baseline was not associated with greater incidence of AF over a 12.9-year median follow-up. Given that GlycA was a composite marker reflective of multiple acute phase proteins, we had hypothesized that it would be a superior marker of predicting AF risk compared to the other inflammatory markers. Rather, we only found IL-6 to be independently associated with incident AF.

We investigated this analysis because previous research has found GlycA to be associated with other diseases with an inflammatory etiology, including auto-immune diseases [30–32] and other types of CVD. In a prior MESA study, higher levels of GlycA were associated with having a greater prevalence of an unfavorable cardiovascular health profile, and with poorer status of many of the individual components of the American Heart Association Life Simple 7 score (i.e. BMI, physical activity, smoking, blood pressure, glucose, cholesterol)–health metrics which are also risk factors for AF [18]. Higher GlycA levels have been linked with subclinical atherosclerosis [30, 33, 34], incident CVD [19–22], and with heart failure with preserved ejection fraction [23]. In a prior MESA study comparing the predictive value of GlycA and other inflammatory biomarkers, GlycA had a significantly predictive value comparable to hsCRP, IL-6, and D-dimer, if not superior, for total death, CVD, chronic inflammatory-related severe hospitalization and death, and total cancer [21]. Thus, we had hypothesized that GlycA might be a superior marker for AF too, although our findings came to a different conclusion. Of note, prior work in MESA has found that GlycA is only modestly to moderately correlated with other markers of inflammation [with Pearson correlation coefficient for GlycA with d-dimer (0.09), IL-6 (0.29), hsCRP (0.47), and fibrinogen (0.49) [18], and thus we had anticipated associations with AF independent of other inflammatory markers.

There is extensive evidence that inflammation plays a key role in the development of AF. The association of some inflammatory biomarkers with AF had been studied, but the relationship between GlycA, specifically, and AF had not been explored before our report. Consistent with our results, in a recent Chronic Renal Insufficiency Cohort (CRIC) study that assessed the association between common inflammatory biomarkers (hsCRP, fibrinogen, TNF- α, and IL-6) and AF, only plasma IL-6 level was significantly associated with both the presence of AF at baseline and new-onset of AF in chronic kidney disease patients [35]. A prior MESA study that evaluated the prognostic value of IL-6 for various CVD outcomes found the association of IL-6 with AF appeared greater among statin users compared to non-users [36]. In contrast to our findings, in that analysis there was no longer a statistically significant association of IL-6 with AF in multivariable-adjusted models, but differences between that study and ours may be their stratification by statin use at baseline which reduced statistical power, and we additionally adjusted for multiple inflammatory markers including GlycA which was not included in their models [36].

Other inflammatory markers have been linked to AF in prior work. One study examined the relationship between collagen biomarkers and AF and demonstrated that high levels of plasma collagen biomarkers were also associated with excess risk for AF [37]. Alegret et al showed that CRP and C-C Motif Chemokine Ligand 2 (CCL2) concentrations were significantly increased in AF patient compared to healthy subjects [38]. However, when patients with paroxysmal AF and permanent AF were separately analyzed, while increase in CCL2 was observed in both subgroups, CRP was only elevated in those with the permanent condition. Further analysis of the genetic variants of CRP and CCL2 did not support strong association of the inflammatory biomarkers and AF, leading to the conclusion that the duration of the episode is important to consider in assessing the role of inflammation, and that elevation of CCL2 is the consequence, not the cause of AF.

Together, these collective studies suggest the relationship between AF and the process of inflammation is multifactorial and complex. High levels of inflammatory biomarkers and inflammatory cells such as neutrophils and lymphocytes have been observed in patients with AF. When local or systemic inflammation occurs, it induces atrial electrical and structural remodeling, which triggers AF. In turn, AF promotes inflammation by mechanisms yet to be understood, and perpetuates the arrhythmia [2]. Markers reflective of acute inflammation vs. chronic inflammation may have differing associations with AF. While inflammation has been implicated in the pathophysiology of AF, and several inflammatory biomarkers have been proposed to be associated with AF, the role of inflammation in guiding AF management has not been well established.

### Strengths and limitations

Our study must be interpreted in the context of several limitations. First, due to our ascertainment methods, paroxysmal cases of AF that were not present at study visits or did not result in hospitalization may have been missed. Second, incident AF cases were ascertained from hospitalization discharge codes, which could have been misclassified. However, these codes have been demonstrated to have adequate positive predictive value for the ascertainment of AF events [39]. Third, while we included several covariates that likely are to influence the development of AF in the statistical models, residual confounding remains a possibility. Fourth, GlycA levels were measured only once at baseline, and thus we were unable to assess change in GlycA levels over time. Previous work has shown that a single baseline measure could accurately capture the short-term inflammatory status over four to 6 months [40]. However, we do not know if the same accuracy holds for longer periods of time.

Despite these limitations, our study had a number of strengths. We used data from a large multiethnic, multiracial cohort of patients without clinical CVD at baseline who were followed longitudinally with a median follow up of 12.9 years to determine development of AF. We were also able to examine multiple biomarkers for inflammation other than our primary biomarker of interest, GlycA, for comparison in the same population. To our knowledge, our study was the first to examine the association of GlycA with AF.

## Conclusions

In summary, we found that elevated GlycA levels at baseline were not associated with higher risk of incident AF, but IL-6 levels were. This suggests that GlycA is not a prediction marker for AF. Further research is required to understand why the inflammatory marker IL-6, but not GlycA, was associated with AF. Several inflammatory biomarkers have been proposed to be associated with AF, but due to the complex pathophysiology of AF each biomarker’s predictive value in guiding AF management has not been well established. Nevertheless, it is undeniable that inflammation contributes to the pathological process of AF. A better understanding of inflammatory markers related to AF improves understanding of the pathophysiology of the disease as well as its risk prediction.

## Supporting information

S1 Table Baseline characteristics of participants by GlycA quartiles. (DOCX)

S2 Table Incidence rates (95% CI) and hazard ratios (95% CI) for the association of GlycA with incident atrial fibrillation, adjusted for incident heart failure and coronary heart disease. (DOCX)
