# Supplementary material for: The association of novel inflammatory marker GlycA and incident atrial fibrillation in the Multi-Ethnic Study of Atherosclerosis (MESA)
Source: PLoS One. 2021 Mar 25;16(3):e0248644. doi: 10.1371/journal.pone.0248644 (PMC7993599; doi:10.1371/journal.pone.0248644)
Supplement: S1 Table — (DOCX) [file pone.0248644.s001.docx]

| **S1 Table. Baseline Characteristics of Participants by GlycA Quartiles, Multi-Ethnic Study of Atherosclerosis (2000-2015)** | | | | | |
| --- | --- | --- | --- | --- | --- |
|  | Quartile 1 | Quartile 2 | Quartile 3 | Quartile 4 | P-value |
|  | n = 1,670 | n = 1,661 | n = 1,630 | n = 1,641 |  |
| ^a^ Age, years | 61 (10) | 62 (10) | 62 (10) | 62 (10) | 0.003 |
| < 65 years | 995 (60%) | 916 (55%) | 888 (54%) | 940 (57%) | 0.01 |
| ≥ 65 years | 675 (40%) | 745 (45%) | 742 (46%) | 701 (43%) |  |
| Sex |  |  |  |  |  |
| Men | 1,033 (62) | 876 (53%) | 720 (44%) | 489 (30%) | < 0.001 |
| Women | 637 (38%) | 785 (47%) | 910 (56%) | 1,152 (70%) |  |
| Race/ethnicity |  |  |  |  |  |
| White | 614 (37%) | 680 (41%) | 600 (37%) | 647 (39%) |  |
| Chinese- American | 327 (20%) | 224 (13%) | 163 (10%) | 77 (5%) | < 0.001 |
| Black | 431 (26%) | 402 (24%) | 473 (29%) | 498 (30%) |  |
| Hispanic | 298 (18%) | 355 (21%) | 394 (24%) | 419 (26%) |  |
| Education |  |  |  |  |  |
| ≥ Bachelor's degree | 736 (44%) | 626 (38%) | 543 (33%) | 430 (26%) | < 0.001 |
| < Bachelor’s degree | 934 (56%) | 1,035 (62%) | 1,087 (67%) | 1,211 (74%) |  |
| ^a^ BMI, kg/m^2^ | 26 (5) | 28 (5) | 29 (5) | 30 (6) | < 0.001 |
| Smoking |  |  |  |  |  |
| Current smoker | 145 (9%) | 185 (11%) | 226 (14%) | 299 (18%) |  |
| Former smoker | 652 (39%) | 623 (38%) | 590 (36%) | 558 (34%) | < 0.001 |
| Never smoker | 873 (52%) | 853 (51%) | 814 (50%) | 784 (48%) |  |
| ^b^ Pack-years of smoking if >0 | 14 (5-26) | 16 (6-32) | 17 (7-34) | 20 (8-38) | < 0.001 |
| ^b^ Physical activity MET-minutes/week | 4,298  (2,265-8,340) | 4,118 (2,010-7,650) | 4,073  (1,965-7,260) | 3,735  (1,710-6,990) | < 0.001 |
| ^a^ Systolic blood pressure, mmHg | 123 (21) | 126 (21) | 128 (22) | 129 (22) | < 0.001 |
| ^a^ eGFR, ml/min per 1.73m^2^ | 79 (15) | 78 (15) | 77 (17) | 77 (18) | < 0.001 |
| ^a^ Total cholesterol, mg/dL | 186 (33) | 194 (33) | 196 (35) | 200 (39) | < 0.001 |
| ^a^ HDL-C, mg/dL | 53 (15) | 51 (15) | 50 (15) | 50 (14) | < 0.001 |
| Diabetes mellitus | 134 (8%) | 173 (10%) | 215 (13%) | 298 (18%) | < 0.001 |
| Antihypertensive medication | 449 (27%) | 561 (34%) | 654 (40%) | 771 (47%) | < 0.001 |
| Lipid-lowering medication | 232 (14%) | 239 (14%) | 292 (18%) | 313 (19%) | < 0.001 |
| ^b^ GlycA, μmol/L | 314 (294-327) | 359 (348-368) | 397 (386-407) | 452 (435-480) | < 0.001 |
| Atrial fibrillation |  |  |  |  |  |
| Yes | 213 (13%) | 226 (14%) | 228 (14%) | 202 (12%) | 0.47 |
| No | 1,457 (87%) | 1,435 (86%) | 1,402 (86%) | 1,439 (88%) |  |
| ^b^ hsCRP, mg/L | 0.85  (0.44-1.64) | 1.45  (0.75-2.88) | 2.43  (1.13-4.63) | 4.69  (2.43-9.75) | < 0.001 |
| ^b^ IL-6 pg/mL | 0.87  (0.59-1.40) | 1.05  (0.72-1.65) | 1.29  (0.87-1.94) | 1.66  (1.15-2.54) | < 0.001 |
| ^b^ Fibrinogen, mg/dL | 300  (268-337) | 327  (291-370) | 351  (311-396) | 386  (339-443) | < 0.001 |
| Abbreviations: BMI, body mass index; MET, metabolic equivalent of task; eGFR, estimated glomerular filtration rate; HDL-C, high-density lipoprotein cholesterol; hsCRP, high-sensitivity C-reactive protein; IL-6, interleukin-6.  ^a^ Data are presented as mean (standard deviation) for continuous variables and as count (percentages) for categorical variables, unless otherwise specified.  ^b^ Data are presented as median (IQR). | | | | | |
