# Supplementary material for: The association of novel inflammatory marker GlycA and incident atrial fibrillation in the Multi-Ethnic Study of Atherosclerosis (MESA)
Source: PLoS One. 2021 Mar 25;16(3):e0248644. doi: 10.1371/journal.pone.0248644 (PMC7993599; doi:10.1371/journal.pone.0248644)
Supplement: S2 Table — (DOCX) [file pone.0248644.s002.docx]

| **S2 Table. Incidence rates (95% CI) and Hazard ratios (95% CI) for the Association of GlycA with Incident Atrial Fibrillation, Adjusted for Incident Heart Failure and Coronary Heart Disease: The Multi-Ethnic Study of Atherosclerosis (2000-2015)** | | | | | |
| --- | --- | --- | --- | --- | --- |
| GlycA in μmol/L  [Median (IQR)] | Quartile 1  [314 (294-327)] | Quartile 2  [359 (348-368)] | Quartile 3  [397 (386-407)] | Quartile 4  [452 (435-480)] | Per 1 SD (62 μmol/L) increment |
| N | 1,670 | 1,661 | 1,630 | 1,641 | 6,602 |
| Cases | 213 | 226 | 228 | 202 | 869 |
| Incidence rates† | 11.2 (9.8-12.8) | 12.0 (10.5-13.7) | 12.4 (10.9-14.1) | 11.2 (9.8-12.9) | 11.7 (11.0-12.5) |
| Hazard Ratios* | 1 (reference) | 0.92 (0.76-1.11) | 0.92 (0.75-1.12) | 0.86 (0.69-1.07) | 0.96 (0.89-1.04) |
| Abbreviation: CI, confidence interval; SD, standard deviation  † Incidence rates reported are per 1000 person-years.  *Model: adjusted for age, sex, race/ethnicity, MESA site, education, health insurance, BMI, smoking status, pack-years of smoking, physical activity, systolic blood pressure, use of antihypertensive medication, total cholesterol, HDL-cholesterol, use of lipid-lowering medication, diabetes, eGFR, incident heart failure and incident coronary heart disease. | | | | | |
